# Psychosocial Determinants of Occupational Health Through the Lenses of Gender Identity and Sexual Orientation

**DOI:** 10.3390/bs15020234

**Published:** 2025-02-18

**Authors:** António Oliveira, Iara Teixeira, Felipe Alckmin-Carvalho, Henrique Pereira

**Affiliations:** 1 Department of Psychology and Education, Faculty of Social and Human Sciences, University of Beira Interior, Pólo IV, 6200-209 Covilhã, Portugal; antonio.oliveira@ubi.pt (A.O.); iara.teixeira@ubi.pt (I.T.); felipe.carvalho@ubi.pt (F.A.-C.); 2 Campus de Gualtar, University of Minho, 4710-057 Braga, Portugal; 3 Research Center in Sports Sciences, Health Sciences and Human Development (CIDESD), 5001-801 Vila Real, Portugal; 4 RISE-Health, Department of Psychology and Education, Faculty of Social and Human Sciences, University of Beira Interior, Pólo IV, 6200-209 Covilhã, Portugal

**Keywords:** occupational health, gender identity, sexual orientation, psychosocial determinants, homonegativity, sexual-related stigma, gender inequality

## Abstract

Understanding the determinants of the occupational health of specific populations and their work-related vulnerabilities is important for developing more effective psychosocial interventions. The aims of this study were (1) to explore differences in occupational health between groups of men versus women and heterosexual versus LGBTQIA+ individuals living in Portugal; (2) to assess whether belonging to LGBTQIA+ groups or being a woman predicts worse occupational health; and (3) to evaluate differences in absenteeism frequency and possible predictors based on sexual orientation and gender. This cross-sectional quantitative study involved 577 participants living and working in Portugal (mean age: 41.62 years, SD = 11.41). To assess occupational health, we used the Copenhagen Psychosocial Questionnaire (COPSOQ III—Middle Version). Women and LGBTQIA+ individuals reported poorer occupational health compared to men and heterosexual individuals, although greater discrepancies were observed in LGBTQIA+ individuals, especially in mental health indicators. Gender and sexual orientation were found to have modest but statistically significant effects on occupational health. The absenteeism frequency was higher among women. Our data suggest improvements in terms of women’s occupational health in Portugal, but not gender equality, which indicates that there are still points to be improved. These findings also suggest the persistence of stigma related to sexual diversity and its effects on the occupational health of LGBTQIA+ and underscore the need for more inclusive workplace policies in Portugal.

## 1. Introduction

Work has historically been essential for human development, providing sustenance and opportunities for personal fulfillment. However, precarious working conditions can lead to significant physical and mental health problems. Occupational health refers to the state of complete physical, mental, and social well-being of workers, not merely the absence of disease or infirmity. It aims to promote and maintain the highest degree of physical, mental, and social well-being among workers in all occupations, in addition to preventing work-related accidents or diseases (WHO, 1995). Historically, occupational health research focused on physical and environmental aspects to protect workers from hazards (Sorensen et al., 2021). However, in recent decades, work-related illnesses, especially those related to psychosocial risks, have increased exponentially (Di Tecco et al., 2023). These risks include tangible factors like workload, and intangible ones, like autonomy and social support (Cox et al., 2000; Bakker & Demerouti, 2017). The International Labour Organization (ILO, 1984) notes that psychosocial risks arise from complex interactions between workers and their environments, affecting performance, job satisfaction, and overall health. In recent years, it has become clear that in industrialized nations, the nature of workplace risks has changed. While physical hazards, such as those related to the working environment, used to be the primary concern, the focus has now shifted more toward psychosocial risks. These include factors like time constraints, the need to multitask, the overall intensity of work, and bullying and pressuring employees for results (Rugulies, 2019).

International statistics highlight the severe impact of working conditions on health and the global economy. The ICOH Congress (2022) reports approximately 2.9 million work-related illnesses and deaths annually worldwide, along with 402 million non-fatal injuries resulting in at least four days of absence. Prolonged exposure to long working hours and substances like particles, gases, and fumes are the primary causes of mortality, leading to thousands of deaths. Economically, occupational accidents and diseases represent an annual loss of 5.4% of the global GDP (ICOH Congress, 2022). Furthermore, more recently, psychosocial determinants^1^ of occupational health have been investigated. A comprehensive study developed by the European Agency for Safety and Health at Work (EU-OSHA, 2022) involving over 27,000 salaried workers from all EU countries, as well as Iceland and Norway, revealed alarming results on workplace mental health. The authors found that 27% of European workers experience stress, anxiety, or depression related to or exacerbated by work. Irregular work schedules and high workplace pressure were identified as some of the most harmful psychosocial factors.

The social and legal framework for work in Portugal has evolved to provide greater protection against discrimination based on sexual orientation, gender identity and expression, and gender characteristics. Law No. 93/2017 establishes a legal framework to prevent and combat discrimination based on race and ethnic origin, nationality, and descent, and promotes a more inclusive work environment (Diário da República, 2017). However, additional legislation and public policies have been implemented to ensure specific protection for LGBTQIA+ people in the workplace. The Commission for Citizenship and Gender Equality (CIG) plays a crucial role in promoting measures to combat discrimination and protect the rights of LGBTQIA+ individuals in the workplace. In the area of occupational health, LGBTQIA+ workers face specific challenges that affect their well-being. Studies show that more than half of LGBT people and about 70% of transgender people have reported experiencing harassment or discrimination at work, which negatively affects their mental health (União Geral de Trabalhadores [UGT], 2020). To address these challenges, the Ministry of Health has established a monitoring group to implement the Health Strategy for LGBTI+ People, strengthen inclusive health policies, and reduce barriers to accessing health services (Serviço Nacional de Saúde [SNS], 2023). Therefore, implementing effective occupational health policies that address the specific needs of LGBTI+ workers is essential to promoting more inclusive and healthy workplaces in Portugal.

A study by Perista et al. (2016) revealed significant exposure to physical and psychosocial risks in positions that involve frequent social interactions, especially in emotionally stressful situations, since these assignments are highly demanding in terms of socioemotional skills, such as self-control, problem-solving ability, communication skills, and empathy. In this same study, although the self-reported levels of harassment, bullying, and violence were relatively low, the authors suggest this may be due to underreporting and underestimation (Perista et al., 2016).

These findings highlight the need to examine how psychosocial risk factors affect various demographic groups differently, such as gender and sexual minorities. Gender, as a social and cultural construct, plays a significant role in shaping individual and collective experiences in the workplace (Ridgeway, 2011; C. Santos & Hilal, 2017; Thistle, 2006; Dernberger & Pepin, 2020). Gender expectations and norms influence professional opportunities, stress levels, and social interactions in the work environment (Eagly & Wood, 2012). Studies show that women and LGBTQIA+ individuals face unique and additional challenges, including pay inequality, a lack of representation in leadership positions, social discrimination, stigma, prejudice, microaggressions, and higher exposure to harassment (ILO, 2016; EIGE, 2021; Pereira & Monteiro, 2017; Oliveira et al., 2024). These gender dynamics not only affect the mental and physical health of these workers but also impact organizational productivity and well-being (Cordero et al., 2024; Reichelt et al., 2020). For instance, the underrepresentation of women in leadership roles and their concentration in lower-paid, less prestigious jobs exacerbate their vulnerability to adverse psychosocial conditions (Lundberg, 2018). Additionally, the accumulation of tasks and the need to balance work and family responsibilities, such as domestic and childcare activities, increases stress levels among women (Cordero et al., 2024; da Silva et al., 2021). Furthermore, several studies indicate that gender inequalities in social and work terms have been worsened by the COVID-19 pandemic, especially in low- and middle-income countries (Reichelt et al., 2020; Alon et al., 2020).

Regarding sexual minorities, studies show a positive association between interpersonal discrimination and negative health outcomes among LGBTQIA+ individuals (De Oliveira Paveltchuk & Callegaro Borsa, 2019; Oliveira et al., 2024). The workplace environment and climate can significantly influence the mental health outcomes of LGBTQIA+ workers (Pereira & Monteiro, 2017; Pereira & Costa, 2016). Studies in various countries have consistently shown that LGBTQIA+ individuals tend to have less favorable mental health outcomes compared to their cisgender heterosexual colleagues (De Oliveira Paveltchuk & Callegaro Borsa, 2019; da Silva et al., 2021; Alibudbud, 2023; Oliveira et al., 2024).

Meyer’s (2003) theory of minority stress provides a valuable framework for understanding this disparity. According to this theory, sexual minorities experience elevated levels of stress due to chronic exposure to stigma, discrimination, and prejudice. These stressors are cumulative and often occur in multiple domains of life, including the workplace. Such stress can lead to significant mental health challenges, as minority stress impairs coping mechanisms and social support, exacerbating psychosocial risks and increasing vulnerability to conditions such as anxiety, depression, and burnout. The theory also highlights that the stress experienced by LGBTQIA+ people is not only a result of overt discrimination, but also of internalized stigma and expectations of rejection, which can influence their behavior and interactions at work.

Therefore, attention to the mediating role of sexual and gender minority status in the association between work-related occupational health measures and psychosocial risks and preventive factors is essential, both for humanitarian and economic reasons. An organizational environment that supports these populations can enhance their job satisfaction (Velez et al., 2013), and policies that promote a supportive environment for minorities can be a competitive advantage for organizations (Trau & Härtel, 2007). This has significant implications for health, social, and work-related interventions, as well as policy measures, especially in Portugal, where the literature is limited (Mendes & Pereira, 2021), discriminatory workplace experiences are common (Beatriz & Pereira, 2022), and psychological distress significantly predicts lower occupational health and well-being (Pereira et al., 2022).

In this context, this study proposes a comprehensive quantitative and cross-sectional examination of the work environment and occupational health among sexual and gender minority workers in Portugal. The aims of this study were (1) to explore differences in occupational health between groups of men versus women and heterosexual versus LGBTQIA+ living in Portugal; (2) to assess whether belonging to LGBTQIA+ groups or being a woman predicts worse occupational and mental health scores; and (3) to evaluate possible predictors of absenteeism among participants.

This study proposes the following hypotheses: (1) LGBTQIA+ and women workers exhibit lower levels of occupational health and well-being related to work and lower levels of mental health, compared to heterosexual individuals and men; (2) belonging to the LGBTQIA+ community or being a woman predicts lower scores in occupational health and mental well-being, with these groups exhibiting a higher risk of mental health issues compared to heterosexual men; and (3) there will be a higher level of absenteeism among LGBTQIA+ and women, related to worse mental health indicators.

## 2. Materials and Methods

### 2.1. Study Design

This is a cross-sectional, comparative, and predictive study. Our research was funded by RESTART, a program of the Foundation for Science and Technology, Portugal (grant number: 2023.00018.RESTART).

### 2.2. Sample and Procedures

To determine the appropriate sample size for this study, we conducted a sample size calculation based on statistical criteria to ensure the representativeness and reliability of the results. We used a power approach to define the number of samples needed to detect significant effects, considering a significance level of 5% (α = 0.05) and a minimum statistical power of 95%. Based on these parameters, the calculation indicated a sample size of 385 participants, selected to minimize Type I and II errors and provide a robust foundation for inferential analysis. The final sample consisted of 577 active individuals aged 18 or older, living in Portugal, fluency in reading the Portuguese language and with a formal study or work relationship with an employer or educational institution for at least 6 months. To ensure a representative and diverse sample of LGBTQIA+ participants, we adopted specific inclusion criteria. In addition to the above criteria, participants were required to self-identify as part of the LGBTQIA+ community. There were no geographical restrictions to ensure a wider range of participants.

Anticipating the possibility that some LGBTQIA+ workers might be hidden and difficult to reach, three sampling techniques were used: one involving direct contacts with organizations, another through formal/informal associations and groups (such as Dezanove, ILGA-Portugal, Opus Gay, and other community groups), and a third using purposive online methodologies to recruit participants through social networks and email lists. We also conducted active outreach on social media, using specific hashtags to reach people from different backgrounds, sexual orientations, and gender identities. To ensure broad diversity, we took steps to include participants from different age groups, gender identities, sexual orientations, and socioeconomic backgrounds. In addition, we aimed to minimize selection bias by providing access to the questionnaire through multiple channels and platforms, including an accessible form, to ensure that people with varying levels of access to technology could participate. These measures were designed to ensure a faithful and inclusive representation of diversity within the LGBTQIA+ community.

Data collection took place between April 2024 and September 2024. For the purposes of this study, participants under the age of 18 and those who were not part of the working population (such as the unemployed and pensioners) were excluded. Subsequently, the questionnaire was distributed via a link sent by email, accompanied by detailed information about the study procedure and the conditions of participation.

### 2.3. Instruments

Sociodemographic Questionnaire: developed by researchers to characterize the sample in this study and provided information such as age, gender, sexual orientation, marital status, academic qualifications, place of residence, socioeconomic status, professional/occupational status, and absences from work in the last year, as well as a self-assessment of physical and mental health, which was answered on a *Likert* scale, ranging from “Very Poor” to “Excellent”.

Copenhagen Psychosocial Questionnaire—COPSOQ III (Middle Version): The third version of the instrument was developed by Burr et al. (2019) and it is a tool developed to assess various psychosocial aspects of the work environment and their impact on workers’ health and well-being. COPSOQ III (*Middle Version*) addresses topics such as psychological demands, control over work, social support, quality of relationships at work, job insecurity, compensation, recognition at work, value conflicts, and organizational justice, as well as aspects related to health and well-being, such as fatigue, musculoskeletal pain, and psychological aspects such as stress and depression. The original instrument was tested in six countries and in most studies, it presented satisfactory psychometric properties (Burr et al., 2019). The Portuguese version of COPSOQ III (*Middle Version*) was developed by Cotrim et al. (2022) and showed excellent reliability (α = 0.913). The questionnaire is structured in 85 items classified in 31 dimensions that allow for a comprehensive analysis of psychosocial conditions in the workplace. All items were measured using a 5-point Likert scale, from “never” to “always” and from “none” to “extremely”. The dimension “self-rated health” is measured with a 5-point scale from “Excellent” to “Poor”.

### 2.4. Data Analysis

Statistical analyses were carried out in SPSS, version 29, and significance was set at 5% (*p* < 0.05). Descriptive analyses included the classification of means, standard deviation, maximum, minimum, and proportion scores for sociodemographic variables, psychosocial risks, and self-assessment of physical and mental health status. Data normality was assessed using the Shapiro–Wilk tests, and homogeneity of variance was assessed using Levene’s test.

After confirming the normality of the data, we conducted a second stage of analysis that involved bivariate and multivariate analyses. Specifically, we utilized Chi-square tests to evaluate the differences between groups for sociodemographic data and Student’s *t*-test to assess the differences between COPSOQ scores. Effect sizes were calculated using Cohen’s d and Cramér’s V, with the following benchmarks: for Cohen’s d, small (0.2–0.5), medium (0.5–0.8), and large (>0.8); and for Cramér’s V, small (<0.30), medium (<0.50), and large (>0.50), as suggested by Cohen (1988).

Additionally, linear regressions were employed to examine the influence of mental health, physical health, stress, sleeping troubles, burnout^2^, and depressive symptoms on absenteeism regarding gender and sexual orientation. For the analysis divided by gender, only cisgender men and women were considered, due to the small number of respondents who identified themselves as transgender, agender and gender fluid, and non-binary.

### 2.5. Ethical Considerations

Ethical principles were respected, and the study was approved by the Ethics Committee of the University of Beira Interior (No. CE-UBI-Pj-2024-022). In our research, we adhered to all ethical criteria to ensure the protection of the participants’ rights, safety, and well-being. We obtained informed consent from all participants, ensuring they were fully informed about the study’s objectives, procedures, risks, and benefits. Our research is scientifically justified, with anticipated benefits outweighing the minimized risks. We rigorously maintained the confidentiality and privacy of participant data. Participant selection was conducted fairly and equitably, without any form of discrimination. We ensured transparency in the dissemination of results, adhering to the ethical principles established by international guidelines, such as the Declaration of Helsinki (WMA, 2013).

## 3. Results

### 3.1. Sociodemographic Information

Table 1 presents the sociodemographic characteristics of a sample of 577 participants, with an average age of 41.62 years (SD = 11.41). Most of the participants are women (73.1%), with smaller representations of men (25.7%), and a minimal portion of non-binary (0.7%), agender (0.2%), and transgender individuals (0.4%). In terms of sexual orientation, most of the respondents identify as heterosexual (84.3%), with minorities identifying as gay (4.6%), lesbian (1.8%), bisexual (6.5%), and other sexual orientations (2.8%). The marital status of the participants is diverse, with a large portion being married to opposite-sex partners (37%), followed by single individuals without a partner (19.6%) and single individuals with a partner (17%). The participants also vary in their educational attainment, with 42.9% holding a master’s degree and 25.8% having a bachelor’s degree. Regarding residence, 43.3% of the participants live in small towns, while 34.7% reside in large cities. Most of the participants classify themselves as belonging to the middle socioeconomic level (59.4%), and 93.4% of the respondents are employed. For the self-rated physical health, 47.1% of the respondents rate their health as good, followed by 34.1%, who rated it as average. Similarly, for the self-rated mental health, the majority of participants also describe their health as good (42.1%) or average (36%). The absenteeism data show that most of the participants had low absenteeism, with 61.3% rarely or never missing work, and 26.3% missing work only a few times. A smaller portion reported more frequent absenteeism, with 9.6% missing work sometimes, 2.5% often, and just 0.2% almost always absent.

To provide a clearer understanding of the sample characteristics, we conducted Chi-square analyses on key sociodemographic variables. These analyses allowed us to explore potential differences between the groups of interest—women versus men (Table 2), and heterosexual versus LGBTQIA+ individuals (Table 3). Given the aims of the study, which involve comparing these groups across various outcomes, this preliminary assessment of sociodemographic distinctions helped contextualize the composition of our sample and ensure a thorough examination of the relevant factors.

The Chi-square analysis revealed significant differences between gender groups across sexual orientation, employment status, and absenteeism (Table 2). We found differences for sexual orientation with a Cramér’s V of 0.271, indicating a small effect size with more women identifying as heterosexual and bisexual than men.

In terms of the employment status, a larger proportion of women were employed compared to men. Men were more likely to be self-employed and employed students than women, who were slightly more represented as students (Cramér’s V of 0.13). In relation to the absenteeism in the last year, women reported more absences than men for this sample (Cramer’s V = 0.14).

Although statistically significant, all the effect sizes were small. The small effect sizes suggest that other variables, such as workplace policies, job types, caring responsibilities, and organizational culture, may play a more important role in generating these patterns.

We used a Chi-squared analysis to test for differences between heterosexual and LGBTQIA+ participants. The LGBTQIA+ participants were analyzed in the same category as there were not enough participants in each subgroup to perform more robust analyses. Significant differences were found between the groups by sexual orientation (Table 3). The marital status distribution differed substantially between these groups. A higher proportion of LGBTQIA+ individuals were single without a partner or with a partner, compared to the heterosexual respondents. The heterosexual individuals, on the other hand, were more likely to be married than the LGBTQIA+ individuals (Cramér’s V = 0.44). For the place of residence (Cramér’s V = 0.13), a larger proportion of the LGBTQIA+ individuals resided in large cities compared to the heterosexual individuals, while the heterosexual individuals were more likely to live in small towns compared to the LGBTQIA+ individuals.

The socioeconomic status also differed between the groups, with a small effect size (Cramér’s V = 0.16). The LGBTQIA+ individuals were more likely to fall into the low-middle and low socioeconomic categories compared to the heterosexual individuals, while the heterosexual individuals were more represented in the medium category. There was a significant difference in the employment status (Cramér’s V = 0.34). The LGBTQIA+ individuals were more likely to be students and emplyed students compared to the heterosexual individuals. On the other hand, the heterosexual individuals were more likely to be employed compared to the LGBTQIA+ individuals.

Finally, for the self-rated mental health, significant differences were observed between the heterosexual and LGBTQIA+ respondents (Cramer’s V = 0.18). More heterosexual individuals rated their mental health as “good”, compared to the LGBTQIA+ individuals, in line with this, more LGBTQIA+ individuals reported their mental health as “poor” compared to the heterosexual individuals.

Although these differences were statistically significant, the effect sizes varied. A larger effect was observed for the marital status (Cramér’s V = 0.44), suggesting notable behavioral differences between the heterosexual and LGBTQIA+ participants. The employment status also showed a moderate effect (Cramér’s V = 0.34). However, the effect sizes for socioeconomic status (Cramér’s V = 0.16), self-rated mental health (Cramér’s V = 0.18), and place of residence (Cramér’s V = 0.13) were small. This suggests that although the differences are measurable, their practical implications may be limited and warrant further investigation in different workplace contexts.

### 3.2. Comparative Analysis of Mean Differences Between Gender and Sexual Orientation Groups

This section will initially present the results pertaining to gender differences, subsequently followed by an examination of the findings related to differences among the sexual orientation groups. These comparisons allow us to investigate potential differences in various work-related psychosocial factors. Significant differences were found between the gender groups for three dimensions: emotional demands, control over working time, and burnout (Table 4).

For emotional demands, the women reported higher levels compared to men (3.41 vs. 3.21; *p* = 0.03, d = 0.20), with a small but significant effect size. Similarly, the women reported less control over their working time (3.69 vs. 3.86; *p* = 0.04, d = 0.19) compared to the men, also with a small but significant effect size. Finally, the women reported higher levels of burnout compared to the men (3.13 vs. 2.94; *p* = 0.03, d = 0.20), again with a small effect size. In all cases, the effect sizes were small but statistically significant, indicating that while gender plays a role in these occupational health outcomes, other factors may have a greater impact.

Table 5 examines the differences between sexual orientations concerning psychosocial dimensions and well-being. For the influence at work, the effect size was small, indicating a significant difference, with the LGBTQIA+ individuals reporting lower levels of influence compared to the heterosexual participants (3.19 vs. 3.45; *p* = 0.01, d = 0.29). Similarly, a small-to-medium effect size was observed for the control over working time, where the LGBTQIA+ individuals reported less control than their heterosexual counterparts (3.47 vs. 3.78; *p* = 0.009, d = 0.32). A moderate effect was noted in the meaning of work, with the LGBTQIA+ participants experiencing less meaning (3.70 vs. 4.09, *p* = 0.002, d = 0.39) compared to the heterosexual individuals. Additionally, commitment to the workplace showed a similar pattern, with a small-to-medium effect size, where the LGBTQIA+ participants demonstrated lower commitment compared to the heterosexual individuals (3.24 vs. 3.58; *p* < 0.001, d = 0.37). For the quality of work, a small-to-medium effect was observed, with the LGBTQIA+ individuals reporting lower quality than the heterosexual individuals (3.61 vs. 3.85; *p* = 0.01, d = 0.30).

Significant differences were also found for the self-rated health, with a medium effect size. The LGBTQIA+ participants rated their health lower compared to the heterosexual participants (3.21 vs. 3.49; *p* < 0.001, d = 0.39). Furthermore, the analysis highlighted that the LGBTQIA+ participants reported higher levels of stress than the heterosexual participants (3.43 vs. 3.08; *p* < 0.001, d = 0.42). Lastly, for depressive symptoms, a small-to-medium effect size (d = 0.35) was identified, with the LGBTQIA+ participants showing higher symptoms compared to the heterosexual participants (2.92 vs. 2.57; *p* = 0.002).

The moderate effect sizes for the meaning of work and self-rated health suggest that these differences may have practical implications for job satisfaction and general well-being. However, further research is needed to explore the extent of these effects. Conversely, the small-to-moderate effect sizes found for control over working time, commitment to work, and quality of work indicate that these differences, although significant, may be affected by other workplace factors such as organizational climate, support networks, and job security.

### 3.3. Regression Analysis of Variable Relationships

Based on the variables that showed significant differences between the groups, we conducted a linear regression analysis to examine the predictive power of gender and sexual orientation on these variables. Table 6 shows the results for gender as a predictor.

The regression analysis indicates that gender had a small but statistically significant negative association with emotional demands (β = −0.094, *p* = 0.023), explaining 0.7% of the variance (R^2^ = 0.007). The relationship between gender and control over working time did not reach statistical significance (*p* > 0.05), explaining only 0.2% of the variance. This suggests that gender alone may not be a strong determinant of the perceived control over work schedules, and other workplace factors—such as job role, industry, and organizational policies—may have a greater influence. However, gender is positively and significantly associated with burnout (β = 0.069, *p* = 0.009), with the model explaining 1.0% of the variance (R^2^ = 0.010). These findings suggest that, in our sample, gender plays a significant role in predicting emotional demands and burnout but not the control over working time.

Regarding sexual orientation as a predictor (Table 7), the sexual orientation was found to have a small but statistically significant effect on several work-related and health outcomes. Specifically, individuals from sexual minority groups reported lower scores in influence at work (β = −0.102, *p* = 0.01), control over working time (β = −0.148, *p* < 0.001), meaning of work (β = −0.122, *p* = 0.004), commitment to the workplace (β = −0.120, *p* = 0.004), quality of work (β = −0.109, *p* = 0.009), and self-rated health (β = −0.161, *p* < 0.001). These results indicate that the LGBTQIA+ workers who answered our questionnaire perceive less autonomy and meaning in their work and experience worse health compared to their heterosexual counterparts.

In terms of health and psychological outcomes, sexual orientation was positively associated with sleeping troubles, stress, and depressive symptoms. The LGBTQIA+ individuals reported higher levels of these symptoms compared to the heterosexual participants. However, the effect sizes were small (R^2^ values ranging from 0.01 to 0.02), indicating that while the differences were statistically significant, their practical impact may be limited and require further exploration in workplace settings.

### 3.4. Analysis of Correlations Between Psychopathology and Absenteeism

We conducted correlation analyses using the variables depression, stress, mental health, physical health, and sleep problems. These analyses were performed separately for women, men, LGBTQIA+ individuals, and heterosexual individuals to evaluate the relationships between psychopathology and absenteeism within each group. For women, absenteeism is negatively correlated with mental health (r = −0.24, *p* < 0.001) and physical health (r = −0.28, *p* < 0.001), and positively associated with stress (r = 0.18, *p* < 0.001), sleeping problems (r = 0.11, *p* < 0.05), burnout (r = 0.18, *p* < 0.001), and depressive symptoms (r = 0.23, *p* < 0.01). For men, absenteeism also shows significant negative correlations with mental health (r = −0.27, *p* < 0.001) and physical health (r = −0.22, *p* < 0.001), but higher correlations with stress (r = 0.27, *p* < 0.001) compared to women, and no significant association with sleeping problems. For heterosexuals, the results mirror those of men and women, with absenteeism negatively associated with mental health (r = −0.25, *p* < 0.001) and physical health (r = −0.27, *p* < 0.001), and positively correlated with stress (r = 0.20, *p* < 0.001), sleeping problems (r = 0.12, *p* < 0.001), burnout (r = 0.20, *p* < 0.001), and depressive symptoms (r = 0.23, *p* < 0.001). However, for the LGBTQIA+ individuals, absenteeism did not show statistically significant correlations with any of the analyzed variables, including mental and physical health, stress, sleep disorders, burnout, and depressive symptoms (*p* > 0.05). This suggests that absenteeism in this group may be influenced by workplace-specific or structural factors that were not captured in this study, such as job security, organizational climate, or coping mechanisms.

For all groups, mental health is strongly positively correlated with physical health and negatively correlated with stress, burnout, and depressive symptoms, though the strength of these correlations varies: For women, mental health is highly negatively correlated with depressive symptoms (r = −0.64, *p* < 0.001) and burnout (r = −0.51, *p* < 0.001). For men, the relationship between mental health and stress is particularly strong (r = −0.68, *p* < 0.001), as is the correlation with depressive symptoms (r = −0.68, *p* < 0.001), indicating a greater impact of mental health on stress and depressive outcomes. Among the heterosexual participants, mental health also shows strong negative correlations with depressive symptoms (r = −0.62, *p* < 0.001) and burnout (r = −0.52, *p* < 0.001), similar to women. For the LGBTQIA+ individuals, mental health has the strongest correlations with depressive symptoms (r = −0.72, *p* < 0.001) and burnout (r = −0.64, *p* < 0.001), suggesting that mental health issues are particularly predictive of depressive symptoms in this group.

Stress is significantly correlated with absenteeism in women (r = 0.18, *p* < 0.001), men (r = 0.27, *p* < 0.001), and heterosexuals (r = 0.20, *p* < 0.001); however, no statistically significant correlation was observed for the LGBTQIA+ individuals. This may suggest that absenteeism in this group is influenced by different stressors or coping mechanisms not captured in the current analysis. Stress also has strong positive correlations with burnout across all the groups, but is particularly high for men (r = 0.68, *p* < 0.001) and heterosexual individuals (r = 0.70, *p* < 0.001), suggesting that stress is more tightly linked to burnout in these groups compared to women (r = 0.67, *p* < 0.001) and LGBTQIA+ individuals (r = 0.60, *p* < 0.001).

Burnout correlates strongly with depressive symptoms across all the groups, with the strongest correlation observed in men (r = 0.70, *p* < 0.001) and LGBTQ individuals (r = 0.72, *p* < 0.001), compared to women and heterosexual individuals, where the correlations are slightly lower (r = 0.66, *p* < 0.001 for both).

Given the observed associations between absenteeism and the studied variables, we conducted a hierarchical linear regression to explore their potential predictive power in absenteeism. The analysis was carried out separately for women, men, heterosexual individuals, and LGBTQIA+ individuals. The hierarchical regression analyses for both men and women indicate that mental health initially plays a significant role in predicting absenteeism, although its effect diminishes in later models. For women, mental health was a significant negative predictor in Model 1 (β = −0.240, *p* < 0.001) and decreased in Model 2 (β = −0.136, *p* = 0.01), becoming non-significant in subsequent models. Similarly, for men, mental health significantly predicted absenteeism in Model 1 (β = −0.270, *p* = 0.001) and Model 2 (β = −0.213, *p* = 0.01), but also became non-significant in later models. Notably, physical health consistently predicted lower absenteeism for women across all the models (e.g., Model 3, β = −0.215, *p* < 0.001), while it did not have a significant effect for men. Additionally, stress, sleeping troubles, burnout, and depressive symptoms were not significant predictors of absenteeism for either gender in any of the models, highlighting the importance of mental and physical health, particularly for women, in determining absenteeism outcomes (Table 8).

In terms of the analysis for the heterosexual and LGBTQIA+ individuals (Table 9), we found that distinct patterns emerged between the heterosexual and LGBTQIA+ individuals regarding the predictors of absenteeism. For the heterosexual individuals, mental health was a significant negative predictor in the initial model (β = −0.251, *p* < 0.001), indicating that better mental health is associated with lower absenteeism, although this effect diminished across the subsequent models. Physical health also consistently predicted lower absenteeism (β = −0.206, *p* < 0.001) throughout all the models. Additionally, stress showed a marginally significant positive effect in one model (β = 0.109, *p* = 0.05). In contrast, for the LGBTQIA+ individuals, none of the variables—including mental health, physical health, stress, sleeping troubles, burnout, and depressive symptoms—emerged as significant predictors of absenteeism across any model. In contrast, for the LGBTQIA+ individuals, none of the analyzed variables were statistically significant predictors of absenteeism in any model. This suggests that absenteeism may be influenced by other workplace-related or contextual factors not included in this study.

## 4. Discussion

This study proposes a comprehensive quantitative and cross-sectional examination of occupational health among sexual and gender minority workers in Portugal. Our primary objectives were to explore differences in occupational health between men and women, as well as between heterosexual and LGBTQIA+ individuals residing in Portugal, and to assess whether belonging to LGBTQIA+ groups or being a woman predicts lower occupational health scores. Additionally, we aimed to investigate differences in absenteeism across these groups and to identify potential predictors of absenteeism.

### 4.1. Ocupational Health Through the Lenses of Gender Identity

When comparing occupational health by gender, our results show slightly lower scores for women across nearly all domains. However, only the domains indicating higher emotional demands^3^, reduced control over work hours, and elevated burnout scores reached statistical significance. These findings support our first hypothesis and align with prior studies examining occupational health through a gender perspective (Cordero et al., 2024; da Silva et al., 2021). Higher emotional demand, reduced perception over time control, and increased burnout among women may be related to additional responsibilities beyond the workplace, including household and childcare tasks that are typically assigned to women due to social expectations and stereotyped views of gender roles (Cordero et al., 2024; Dernberger & Pepin, 2020; Eagly & Wood, 2012).

No significant differences between men and women were observed in the other 28 occupational health domains, suggesting progress toward greater gender equity in Portuguese workplaces. While advancements in task division and occupational health have likely occurred in recent decades, a state of “gender flexibility” rather than equality has been achieved in many Western countries, indicating that ongoing efforts are needed to minimize remaining gender-related discrepancies, even if they are smaller than they were in the past (Dernberger & Pepin, 2020).

A regression analysis was conducted to assess the predictive power of gender on worker health. Our results indicated a modest but statistically significant effect of gender on several work- and health-related outcomes, which contradicts our second hypothesis. We had anticipated a stronger pWhredictive power of gender on the occupational health of the participants evaluated. Specifically, we expected that being a woman would predict lower occupational health scores, a finding that proved to be only partially accurate. While we found significant differences in some occupational health domains—particularly those related to workload and burnout, the effect size was small. This result suggests that gender-based occupational health inequalities in Portugal may be diminishing. Furthermore, the significant differences observed could be related to uncontrolled variables in our study, given the low predictive power of gender in our regression analysis.

It is plausible that job insecurity contributes to slightly lower occupational health scores among women. A large study evaluating job insecurity and mental health in 22,555 workers across 22 European countries including Portugal found significantly higher precarious employment scores for women (mean = 32.31, SD = 0.33) compared to men (mean = 27.32, SD = 0.32), though the differences were smaller in Southern Europe, including Portugal [mean = 28.81 SD = 0.32 for men, mean = 30.24 SD = 0.31 for women] (Padrosa et al., 2022). The authors found significant differences only in the wage disparity domain (mean = 34.17, SD = 0.84 for men vs. mean = 43.83, SD = 0.97 for women), indicating greater income precarity for women, with minimal gender differences in vulnerability factors such as managerial respect, work schedule predictability, exercise of rights, and representativeness (Padrosa et al., 2022). Thus, although studies in developing countries, such as Brazil and Mexico, still report significant gender disparities in worker occupational health (Sousa & Araújo, 2024; Biswas et al., 2021), these differences in Portugal, though persistent, appear to be much less significant.

### 4.2. Occupational Health Through the Lenses of Sexual Orientation

Our analysis of LGBTQIA+ occupational health reveals several specific discrepancies when compared to the heterosexual group. The LGBTQIA+ participants reported significantly lower levels of workplace influence, control over work hours, job meaning, and work commitment. Additionally, they exhibited higher rates of sleep disturbances, depressive symptoms, stress, and self-reported physical health concerns. These findings support our first hypothesis and are consistent with previous Portuguese studies (Mendes & Pereira, 2021; Pereira et al., 2022).

These results also align with findings reported in other Western countries. A recent narrative review by Oliveira et al. (2024) analyzed outcomes of eleven studies on occupational health, psychosocial risks, and preventive factors among LGBTQIA+ workers. Despite changes in legislation and social attitudes in some countries, discrimination against LGBTQIA+ individuals persist in workplace environments, often in the form of microaggressions, negatively impacting well-being, occupational health, and job satisfaction. The review emphasized that LGBTQIA+ individuals face additional psychosocial challenges, including decisions about whether to disclose their gender identity or sexual orientation, often influenced by factors such as the presence of anti-discrimination policies and the degree of institutional support. Oliveira et al. (2024) therefore underscore the need for inclusive and equitable work environments that explicitly support LGBTQIA+ employees and periodically evaluate subtle and less evident forms of discrimination.

A regression analysis was conducted to evaluate the predictive power of sexual orientation on the participants’ occupational health. Our findings indicated a modest but statistically significant effect of sexual orientation on nine work- and health-related outcomes. We had anticipated a stronger predictive power of sexual orientation, especially in mental health domains such as depression, stress, burnout, and sleep problems. Although we found lower occupational health scores for the LGBTQIA+ population, our regression analysis revealed a low predictive power of sexual orientation in this outcome, suggesting that other uncontrolled variables in our study may be responsible for these discrepancies.

The absence of equity and inclusion in the workplace can generate additional stressors for individuals from marginalized groups, such as LGBTQIA+ individuals. These stressors may contribute to higher levels of psychosocial stress, anxiety, depression, and feelings of isolation. Discrimination, prejudice, and exclusion, present in non-inclusive work environments, have the potential to aggravate these problems, compromising the well-being of workers (Cordero et al., 2024; Reichelt et al., 2020; Oliveira et al., 2024). In contrast, an inclusive work environment, which values diversity and promotes equity, can contribute to strengthening the sense of belonging and support among employees, resulting in mental health benefits and a healthier and more productive environment (Oliveira et al., 2024).

Another fundamental factor is the organizational climate and inclusion policies within companies, which, when inadequate, may contribute to exacerbating mental health challenges faced by LGBTQIA+ workers. Research has shown that organizations with inclusive policies and a supportive workplace culture significantly reduce stress levels and improve the psychological well-being of LGBTQIA+ employees (Deloitte, 2023; Oliveira et al., 2024). Furthermore, external support networks, such as family and social support, play a crucial role. LGBTQIA+ individuals with robust external support demonstrate greater resilience and better coping mechanisms in dealing with workplace stressors (Chan et al., 2022). Finally, workplace norms and culture directly impact the comfort and acceptance levels experienced by LGBTQIA+ employees, highlighting that the cultural and social context of a workplace can mitigate or amplify the adverse effects of a non-inclusive environment (Mara et al., 2021).

### 4.3. Absenteeism

Our third objective was to compare the frequency of absenteeism among the analyzed groups and to assess whether the self-reported signs and symptoms of psychopathologies, such as depression, stress, burnout, and sleep problems, could be associated absenteeism. Our results revealed higher absenteeism scores among women but not between LGBTQIA+ individuals, compared to men and heterosexual participants. This result corroborates our hypothesis about greater absenteeism among women, reiterating results from previous studies (Deloitte, 2023; Gonçales & Zanatti, 2023; Velez et al., 2013). However, the similar frequency of absenteeism among LGBTQIA+ and heterosexual individuals contradicts our hypothesis according to which LGBTQIA+ individuals would present higher absenteeism scores. Our initial hypothesis about higher absenteeism among LGBTQIA+ individuals was grounded from the perspective of minority stress (Frost & Meyer, 2023). This theory proposes that people belonging to marginalized or stigmatized groups, such as LGBTQIA+ individuals, face disproportionate levels of stress and have mental health and quality of life indicators lower than those found in individuals not belonging to minority groups, and that these socially produced vulnerabilities increase the risks of psychopathologies (De Oliveira Paveltchuk & Callegaro Borsa, 2019; da Silva et al., 2021; Alibudbud, 2023). Our impression was that greater psychosocial vulnerability might be associated with a higher probability of absenteeism and absences from work for mental health reasons, which was not confirmed by our results.

We suggest that, despite significant mental health challenges, some LGBTQIA+ individuals may find solace in the workplace, using it as a refuge from external stressors. Furthermore, they may engage in overcompensation behaviors to cope with internalized homonegativity. Overcompensation related to low self-esteem and homonegativity may manifest in many ways, such as heightened productivity, perfectionism or strong commitment with work, functioning as a protective mechanism against sexual stigma perceived in their community and self-stigma. However, although the mechanism of overcompensation is associated with high productivity and quality of work (overachieving), at the individual level, it may produce self-esteem that is overly influenced by performance and results, which hinders the formation of a relatively stable positive identity (Bridge et al., 2019, 2022). In addition, overcompensation can make it difficult to identify aspects of internalized homonegativity, which may reduce the likelihood of seeking psychotherapeutic treatment. Finally, the mechanism of overcompensation can jeopardize self-care in order not to compromise the productivity of the individual, who may, for example, continue to work even during periods of physical and mental illness. We believe that these mechanisms can explain, at least partially, why this population did not present higher rates of absenteeism despite reporting elevated levels of occupational health problems. This hypothesis will be investigated in a new qualitative study, currently underway, associated with the same research project from which this article originated.

In addition, we found a significant negative correlation between women’s mental and physical health and absenteeism. We also found significant and positive correlations between stress, sleep disorders, burnout and depressive symptoms, and absenteeism in this group of participants. These results suggest that the overload of Portuguese women with several demands parallel to and additional to work demands can produce overload of responsibilities, stress, and other mental health problems, conditions that increase the probability of absenteeism. These findings highlight the importance of greater equity in the division of tasks among the Portuguese population, the importance of measures to recognize overload among women, and instruments/strategies for the identification and management of this condition and prevention of mental health problems and absenteeism.

From a sexual orientation perspective, absenteeism among heterosexual workers was a significant predictor of depressive symptoms, but for LGBTQIA+ people, this association was not verified. In the comparison between heterosexuals and LGBTQIA+ people, the results do not support our hypothesis, since no significant differences in absenteeism were observed between these groups. Interestingly, in the case of the LGBTQIA+ population, absenteeism did not show a significant correlation with any of the variables analyzed, including mental and physical health, stress, sleep disorders, burnout, and depressive symptoms. This finding suggests that absenteeism in this population may be influenced by factors external to the health variables investigated, indicating that the determinants of this phenomenon may be different for LGBTQIA+ individuals.

In addition to the immediate benefit of inclusive policies, the reduction in absenteeism directly impacts productivity and organizational costs, since prolonged absences represent significant financial losses, as revealed in a recent study of 1292 Canadian workers. The main results of this study revealed that the psychological distress associated with absenteeism generates high annual costs for employers, specifically, $2337 for women and $2796 for men (Gilbert-Ouimet et al., 2024).

Studies indicate that the stress caused by discrimination is one of the main factors for the development of psychopathologies in LGBTQIA+ people, such as anxiety and depression, conditions that affect attendance and performance. By promoting a welcoming and prejudice-free work environment, companies favor the well-being of LGBTQIA+ employees and reduce indirect costs linked to psychopathologies and high turnover, showing that inclusive policies are essential not only for social justice, but also as an economic strategy to optimize resources.

Companies that adopt a culture of mental health support not only contribute to the well-being of their employees, but also become more attractive to talent, making it easier to attract and retain qualified professionals. In addition, by creating a more positive and collaborative work environment, these organizations strengthen employee commitment and motivation, resulting in a more productive and healthier organizational climate. The promotion of mental health in the workplace, as highlighted by the World Health Organization and International Labour Organization (2021), is therefore an effective strategy to improve the overall performance of the company and the well-being of its employees.

### 4.4. Practical Implications

This research highlights the need for workplace interventions that promote inclusion and equity in terms of gender identity and sexual orientation, although our study suggests that this second category requires more concentrated efforts, as they have greater discrepancies in Portugal. Although progress has been made in terms of mitigating intolerance and violence against sexual and gender minorities in the workplace, our study corroborates previous studies and indicates that inequalities based on sexual orientation and gender persist in companies and educational and health institutions. The creation and periodic improvement of affirmative legislation and policies on sexual and gender diversity, developed and sustained by countries, such as the criminalization of homophobia and the explicit valuing of sexual diversity, is an important step towards ensuring the human rights of LGBTQI+ individuals and gender minorities. However, studies indicate that this measure alone does not guarantee the protection of these individuals (Oliveira et al., 2024; Di Marco et al., 2021; Lloren & Parini, 2017; Grenier & Hixson-Vulpe, 2017). There is vast literature indicating the discrepancy between the laws protecting the rights of the LGBTQIA+ population and the attitudes of institutions and society, highlighting the need for continued efforts to safeguard and promote the human rights of this community, which has historically been exposed to stigmatization and various forms of social injustice due to the marginalization of their identities, including in the employment context.

Among the measures recommended for companies and institutions, we highlight the need to ensure representativeness; decision-makers should actively strive to hire individuals of different sexual orientations and gender identities, including transgender individuals and cross-dressers, who still face many difficulties in entering the formal labor market (K. M. D. O. Santos & Oliveira-Silva, 2021; Abreu et al., 2023). It is necessary to make it possible for women, regardless of their sexual orientation, and other LGBTQIA+ individuals to compete on equal terms for management positions, since they remain underrepresented in the power spaces of companies and other institutions. Ensuring the representation of these individuals in these spaces increases the likelihood of inclusive policies being implemented and improves the sense of security and belonging of sexual and gender minorities in companies and institutions. In addition, organizational initiatives to combat heteronormativity and sexism, as well as the implementation and enforcement of anti-discrimination policies, should be explicitly included among companies’ values. Training should be provided on a regular basis, and the appreciation of sexual and gender diversity should form part of diversity and inclusion education programs for company employees, including those in senior positions. Finally, formal channels for reporting cases of homophobia, transphobia or misogyny should be developed, and employees should be made aware of this alternative. To discourage homonegativity, transphobia or misogyny, witnesses to these situations should be encouraged to take a stand, to create an organizational climate of intolerance towards discriminatory behavior.

The creation of psychologically safe work environments, with adequate social support and clear anti-discrimination policies, is essential to improve the mental health and well-being of LGBTQIA+ workers and women. The literature already suggests that organizations that implement such inclusive policies tend to have a more satisfied and productive workforce (Trau & Härtel, 2007). In addition, our study highlights the importance of preventive approaches in the management of workers’ health. Identifying and mitigating the psychosocial risks faced by sexual and gender minorities should be a priority in workplace health policies. The literature indicates that the prevention of psychosocial risks not only improves worker well-being, but also reduces costs related to occupational diseases and absenteeism (Sorensen et al., 2021). Finally, the discussion presented in this study emphasizes that occupational health interventions should be adapted based on the specific needs of different population groups. Implementing strategies that consider the lived experiences of women and LGBTQIA+ individuals in the workplace is not only a matter of social justice but also of organizational efficiency and improved public health.

### 4.5. Limitations and Future Perspectives

Although we have achieved the objectives proposed in our article, our study is not without limitations. The cross-sectional nature of our research prevents the determination of causality between the factors analyzed and the occupational health outcomes. Furthermore, the LGBTQIA+ category encompasses a diversity of identities and experiences and, due to the small sample size for the subgroups, our study treated this population as a single group. However, different subgroups within the LGBTQIA+ community may face different challenges. Future research should analyze each subgroup separately to identify specific vulnerabilities and needs. The data collection via the online questionnaire resulted in the exclusion of individuals without access to the internet or with less technological familiarity. This implies the underrepresentation of a portion of the Portuguese population that lives in a condition of digital exclusion, usually associated with poverty or advanced age. Studies aimed at understanding the determinants of occupational health among this population are necessary. In addition, the occupational health of our participants was assessed based on self-reports, which may be biased by the social desirability and self-assessment capacity of each individual. In addition, in the composition of our sample, we found an underrepresentation of men and individuals with less schooling, which limits the generalization of the results to other populations. We suggest, therefore, the replication of this study with more representative samples of the Portuguese population, in order to increase the external validity of the results. Furthermore, the study being carried out in Portugal implies that the conclusions may not apply to other socio-cultural and legislative contexts in other countries of the European Union. Finally, our study did not include transgender individuals and cross-dressers, which fails to capture the unique occupational health challenges faced by this population, which studies indicate are much more vulnerable to discrimination and prejudice in several countries, including Portugal (Neves et al., 2023). To address this gap, future studies should evaluate the occupational health of these individuals, as well as compare these conditions with other groups, to produce valuable insight that supports public policies sensitive to the nuances and specificities of this population.

## 5. Conclusions

Our study responds to a call for investigation of occupational health inequalities among sexual and gender minorities in Portugal. Our data suggest improvements in terms of women’s occupational health in Portugal, but not gender equality. They indicate that there are still points to be improved. They also suggest the persistence of the inequity of occupational health of LGBTQIA+ individuals and underscore the need for more inclusive workplace policies for sexual minorities in Portugal.

## Figures and Tables

**Table 1:** Sociodemographic characteristics.

Variable	Category	N	%
Gender (*n* = 576)	Woman	421	73.1
	Man	148	25.7
	Non-binary	4	0.7
	Fluid	0	0
	Agender	1	0.2
	Trans man	1	0.2
	Trans woman	1	0.2
Sexual Orientation (*n* = 568)	Heterosexual	479	84.3
	Gay	26	4.5
	Lesbian	10	1.7
	Bisexual	37	6.5
	Pansexual	11	1.9
	Asexual	1	0.2
	Queer	4	0.7
Marital Status (*n* = 576)	Single without a partner	113	19.6
	Single with a partner	98	17
	Married to a same-sex partner	7	1.2
	Married to an opposite-sex partner	213	37
	In a domestic partnership with a same-sex partner	7	1.2
	In a domestic partnership with an opposite-sex partner	98	17
	Divorced/separated from a same-sex partner	4	0.7
	Divorced/separated from an opposite-sex partner	32	5.6
	Widowed from a same-sex partner	1	0.2
	Widowed from an opposite-sex partner	3	0.5
Educational Attainment (*n* = 574)	Up to 9 years of schooling	7	1.2
	Up to 12 years of schooling	75	13.1
	Graduate degree	148	25.8
	Masters degree	246	42.9
	Ph.D. degree	97	16.9
	Other	1	0.2
Place of Residence (*n* = 573)	A small rural area	57	9.9
	A large rural area	69	12
	A small town	248	43.3
	A large city	199	34.7
Socioeconomic Status (*n* = 577)	Low	24	4.2
	Low-middle	115	19.9
	Medium	343	59.4
	Upper-middle	90	15.6
	High	5	0.9
Employment Status (*n* = 577)	Student	38	6.6
	Student, employed	71	12.3
	Self-employed	67	11.6
	Employee	401	69.5
Self-Rated Physical Health (*n* = 577)	Very poor	11	1.9
	Poor	55	9.5
	Average	197	34.1
	Good	272	47.1
	Excellent	42	7.3
Self-Rated Mental Health (*n* = 577)	Very poor	10	1.7
	Poor	72	12.5
	Average	208	36
	Good	243	42.1
	Excellent	44	7.6
Absenteeism (Last Year) (*n* = 551)	Never or almost never	338	61.3
	Few times	145	26.3
	Sometimes	53	9.6
	Often	14	2.5
	Always or almost always	1	0.2

**Table 2:** Sociodemographic characteristics by gender.

		Gender				
Variable	Category	Women (%)	Men (%)	Chi-Square (χ²)	*p*	Cramér’s V
Sexual Orientation (*n* = 568)				207.858	<0.001 **	0.271
	Heterosexual	88.2	76.9			
	Gay	-	17			
	Lesbian	2.4	-			
	Bisexual	6.8	4.8			
	Pansexual	1.9	0.7			
	Asexual	0.2	-			
	Queer	0.5	0.7			
Marital Status (*n* = 576)				25.879	0.99	-
	Single without a partner	18.8	20.9			
	Single with a partner	17.1	16.9			
	Married to a same-sex partner	1	2			
	Married to an opposite-sex partner	37.3	37.2			
	In a domestic partnership with a same-sex partner	0.7	2.7			
	In a domestic partnership with an opposite-sex partner	17.1	16.2			
	Divorced/separated from a same-sex partner	0.7	0.7			
	Divorced/separated from an opposite-sex partner	6.4	3.4			
	Widowed from a same-sex partner	0.2	-			
	Widowed from an opposite-sex partner	0.7	-			
Educational Attainment (*n* = 573)				20.188	0.73	-
	Up to 9 years of schooling	0.5	3.4			
	Up to 12 years of schooling	13.6	11.6			
	Graduate degree	25.3	27.2			
	Masters degree	44.2	39.5			
	Ph.D. degree	16.2	18.4			
	Other	0.2	-			
Place of Residence (*n* = 572)				10.635	0.77	-
	A small rural area	10.7	7.6			
	A large rural area	11.4	14.5			
	A small town	44.8	39.3			
	A large city	33.1	38.6			
Socioeconomic Status (*n* = 576)				27.690	0.11	-
	Low	3.8	5.4			
	Low-middle	19.2	20.3			
	Medium	62.5	53.4			
	Upper-middle	14.3	18.2			
	High	0.2	2.7			
Employment Status (*n* = 576)				31.538	0.007 *	0.13
	Student	5.9	7.4			
	Student, employed	11.4	14.9			
	Self-employed	10.7	14.2			
	Employee	72	63.5			
Self-Rated Physical Health (*n* = 569)				26.252	0.15	-
	Very poor	1.4	3.4			
	Poor	9.7	7.4			
	Average	34.0	35.1			
	Good	46.8	48.6			
	Excellent	8.1	5.4			
Self-Rated Mental Health (*n* = 569)				26.441	0.15	-
	Very poor	1.4	2.7			
	Poor	12.6	11.5			
	Average	35.9	36.5			
	Good	43.0	40.5			
	Excellent	7.1	8.8			
Absenteeism (Last Year) (*n* = 543)				45.543	<0.001 **	0.14
	Never or almost never	59.9	65.3			
	Few times	27.3	24.3			
	Sometimes	10.0	8.3			
	Often	2.8	1.4			
	Always or almost always	-	0.7			

* *p* < 0.05; ** *p* < 0.001.

**Table 3:** Sociodemographic characteristics by sexual orientation.

Sexual Orientation						
Variable	Category	Heterosexual (%)	LGBTQIA+ (%)	Chi- Square (χ²)	*p*	Cramér’s V
Marital Status (*n* = 568)				110.375	<0.001 **	0.44
	Single without a partner	15.9	39.3			
	Single with a partner	14.6	30.3			
	Married to a same-sex partner	-	5.6			
	Married to an opposite-sex partner	42.8	-			
	In a domestic partnership with a same-sex partner	-	6.7			
	In a domestic partnership with an opposite-sex partner	18.2	10.1			
	Divorced/separated from a same-sex partner	0.6	1.1			
	Divorced/separated from an opposite-sex partner	6.5	-			
	Widowed from a same-sex partner	0.2	-			
	Widowed from an opposite-sex partner	0.6	-			
Educational Attainment (*n* = 565)				5.678	0.33	-
	Up to 9 years of schooling	1.5	-			
	Up to 12 years of schooling	11.6	19.1			
	Graduate degree	26.7	21.3			
	Masters degree	42.9	42.7			
	Ph.D. degree	17.2	16.9			
	Other	0.2	-			
Place of Residence (*n* = 564)				9.978	0.01 *	0.13
	A small rural area	10.1	9.1			
	A large rural area	13.0	6.8			
	A small town	44.7	35.2			
	A large city	32.1	48.9			
Socioeconomic Status (*n* = 577)				14.904	0.005 *	0.16
	Low	3.5	5.6			
	Low-middle	17.5	32.6			
	Medium	62.8	42.7			
	Upper-middle	15.2	18.0			
	High	0.8	1.1			
Employment Status (*n* = 568)				67.493	<0.001 **	0.34
	Students	4.0	21.3			
	Student, employed	9.4	28.1			
	Self-employed	12.5	5.6			
	Employee	74.1	44.9			
Self-Rated Physical Health (*n* = 568)				3.045	0.55	-
	Very poor	1.9	2.2			
	Bad	8.8	13.5			
	Average	33.8	34.8			
	Good	47.8	44.9			
	Excellent	7.7	4.5			
Self-Rated Mental Health (*n* = 568)				19.209	0.001 **	0.18
	Very poor	1.7	2.2			
	Poor	11.1	21.3			
	Average	33.8	47.2			
	Good	45.1	25.8			
	Excellent	8.4	3.4			
Absenteeism (Last Year) (*n* = 551)				5.776	0.21	-
	Never or almost never	62.9	52.3			
	Few times	25.1	33.0			
	Sometimes	9.0	13.6			
	Often	2.8	1.1			
	Always or almost always	0.2	-			

* *p* < 0.05; ** *p* < 0.001.

**Table 4:** Mean scores by gender (*n* = 569) in psychosocial dimensions using COPSOQ III.

			Gender						
	Total (*n* = 577)		Women (*n* = 421)		Men (*n* = 148)				
Dimensions	M	SD	M	SD	M	SD	*t*	*p*	d
Quantitative Demands	2.79	0.86	2.78	0.89	2.82	0.78	−0.412	0.68	-
Work Pace	3.18	0.95	3.19	0.94	3.15	0.95	0.442	0.65	-
Cognitive Demands	3.78	0.70	3.79	0.70	3.79	0.73	0.048	0.96	-
Emotional Demands	3.35	1.00	3.41	0.98	3.21	1.03	2.100	0.03 *	0.20
Influence at Work	3.41	0.89	3.39	0.88	3.46	0.91	−0.802	0.42	-
Development Possibilities	4.02	0.88	4.03	0.87	3.99	0.94	0.518	0.60	-
Control Over Working Time	3.73	0.91	3.69	0.93	3.86	0.85	−2.008	0.04 *	0.19
Meaning of Work	4.03	0.91	4.07	0.90	3.94	0.95	1.479	0.14	-
Commitment to the workplace	3.53	0.87	3.53	0.87	3.53	0.87	0.092	0.92	-
Predictabiity	3.33	1.00	3.35	0.99	3.31	1.04	0.443	0.65	-
Recognition	3.64	1.02	3.65	1.01	3.65	1.05	0.053	0.95	-
Role Clarity	4.04	0.80	4.05	0.77	4.00	0.89	0.647	0.51	-
Role Conflicts	2.92	0.79	2.90	0.80	2.99	0.75	−1.235	0.21	-
Quality of Leadership	3.34	0.98	3.32	0.97	3.43	1.00	−1.185	0.23	-
Social Support from Colleagues	3.62	0.86	3.66	0.86	3.51	0.84	1.726	0.08	-
Social Support from Supervisors	3.13	1.03	3.13	1.02	3.16	1.07	−0.304	0.76	-
Sense of Community at Work	3.79	0.89	3.80	0.90	3.78	0.84	0.245	0.78	-
Job Insecurity	2.64	1.21	2.65	1.19	2.56	1.26	0.787	0.43	-
Insecurity over Working Conditions	2.05	0.91	2.05	0.87	2.06	0.99	−0.072	0.94	-
Quality of Work	3.81	0.77	3.81	0.76	3.80	0.82	0.142	0.88	-
Horizontal Trust	2.59	0.77	2.57	0.76	2.62	0.78	−0.789	0.431	-
Vertical Trust	3.64	0.87	3.68	0.82	3.56	0.97	1.311	0.19	-
Organizational Justice	3.29	0.87	3.26	0.84	3.39	0.98	−1.425	0.15	-
Work–Life Conflict	3.05	0.99	3.04	0.99	3.08	0.99	−0.480	0.631	-
Job Satisfaction	3.49	0.83	3.47	0.81	3.54	0.89	−0.897	0.37	-
Self-Rated Health	3.45	0.73	3.46	0.73	3.43	0.74	0.142	0.88	-
Self-Efficacy	3.86	0.64	3.87	0.64	3.85	0.60	0.311	0.75	-
Sleeping Troubles	2.83	0.99	2.84	0.98	2.80	1.00	0.438	0.662	-
Burnout	3.08	0.95	3.13	0.92	2.94	1.00	2.084	0.03 *	0.20
Stress	3.12	0.85	3.14	0.83	3.09	0.89	0.562	0.57	-
Depressive Symptoms	2.62	0.96	2.61	0.94	2.62	1.02	−0.110	0.91	-

* *p* < 0.05.

**Table 5:** Mean scores by sexual orientation (n = 568) in psychosocial dimensions and health and well-being using COPSOQ III.

	Sexual Orientation						
	Heterosexual (*n* = 479)		LGBTQIA+ (*n* = 89)				
Dimensions	M	SD	M	SD	*t*	*p*	d
Quantitative Demands	2.79	0.85	2.79	0.91	0.05	0.96	-
Work Pace	3.18	0.94	3.20	0.96	−0.116	0.90	-
Cognitive Demands	3.80	0.69	3.73	0.74	0.846	0.39	-
Emotional Demands	3.37	0.97	3.29	1.14	0.656	0.51	-
Influence at Work	3.45	0.89	3.19	0.87	2.482	0.01 *	0.29
Development Possibilities	4.02	0.87	4.07	0.95	−0.570	0.56	-
Control Over Working Time	3.78	0.87	3.47	1.05	2.655	0.009 *	0.32
Meaning of Work	4.09	0.86	3.70	1.10	3.217	0.002 *	0.39
Commitment to the Workplace	3.58	0.84	3.24	0.98	3.434	<0.001 **	0.37
Predictabiity	3.37	0.99	3.15	1.03	1.846	0.065	-
Recognition	3.67	1.01	3.52	1.08	1.214	0.225	-
Role Clarity	4.06	0.80	3.93	0.82	1.409	0.159	-
Role Conflicts	2.90	0.76	3.04	0.89	−1.379	0.171	-
Quality of Leadership	3.37	0.97	3.18	1.02	1.704	0.08	-
Social Support from Colleagues	3.60	0.86	3.74	0.83	−1.421	0.156	-
Social Support from Supervisors	3.14	1.03	3.14	1.02	−0.008	0.99	-
Sense of Community at Work	3.81	0.89	3.68	0.88	1.249	0.21	-
Job Insecurity	2.62	1.19	2.74	1.31	−0.852	0.39	-
Insecurity over Working Conditions	2.07	0.91	1.98	0.92	0.477	0.400	-
Quality of Work	3.85	0.75	3.61	0.83	2.521	0.01 *	0.30
Horizontal Trust	2.59	0.77	2.62	0.79	−0.376	0.70	-
Vertical Trust	3.66	0.84	3.47	0.99	1.707	0.09	-
Organizational Justice	3.30	0.86	3.23	0.96	0.691	0.49	-
Work–Life Conflict	3.06	0.99	3.04	0.99	0.163	0.87	-
Job Satisfaction	3.50	0.83	3.37	0.84	1.351	0.17	-
Self-Rated Health	3.49	0.72	3.21	0.73	3.300	<0.001 **	0.39
Self-Efficacy	3.87	0.61	3.75	0.76	1.451	0.15	-
Sleeping Troubles	2.81	0.97	3.03	1.00	−1.979	0.04 *	0.22
Burnout	3.06	0.94	3.15	1.02	−0.747	0.45	-
Stress	3.08	0.85	3.43	0.81	−3.592	<0.001 **	0.42
Depressive Symptoms	2.57	0.94	2.92	1.03	−3.132	0.002 *	0.35

* *p* < 0.05; ** *p* < 0.001.

**Table 6:** Linear regression with gender as a predictor of emotional demands, control over working time, and burnout.

Variable	β	R²	F (df)	*p*
Emotional Demands	−0.094	0.007	5.163 (1.574)	0.023 *
Control Over Working Time	0.065	0.002	2.419 (1.574)	0.12
Burnout	0.069	0.010	6.819 (1.574)	0.009 *

* *p* < 0.005.

**Table 7:** Linear regression with sexual orientation as predictor of influence at work, control over working time, meaning of work, commitment to the workplace, quality of work, self-rated health, sleeping troubles, stress, and depressive symptoms.

Variable	β	R²	F (df)	*p*
Influence at Work	−0.102	0.01	5.951 (1.566)	0.01 *
Control Over Working Time	−0.148	0.02	12.613 (1.566)	<0.001 **
Meaning of Work	−0.122	0.01	8.483 (1.566)	0.004 *
Commitment to the Workplace	−0.120	0.01	8.232(1.566)	0.004 *
Quality of Work	−0.109	0.01	6.775 (1.566)	0.009 *
Self-Rated Health	−0.161	0.02	15.092 (1.566)	<0.001 **
Sleeping Troubles	0.100	0.01	5.679 (1.566)	0.01 *
Stress	0.168	0.02	16.339 (1.566)	<0.001 **
Depressive Symptoms	0.155	0.02	13.904 (1.566)	<0.001 **

* *p* < 0.05; ** *p* < 0.001.

**Table 8:** Hierarchical linear regression with mental health, physical health, stress, sleeping troubles, burnout, and depressive symptoms as predictors of absenteeism regarding gender.

**Women**	**Model 1**			**Model 2**			**Model 3**			**Model 4**			**Model 5**			**Model 6**		
**Absenteeism**	**β**	***t***	***p***	**β**	***t***	***p***	**β**	***t***	***p***	**β**	***t***	***p***	**β**	***t***	***p***	**β**	***t***	***p***
Mental Health	−0.240	−4.921	<0.001 **	−0.136	−2.483	0.01 *	−0.095	−1.571	0.11	−0.101	−1.646	0.10	−0.095	−1.519	0.13	−0.057	−0.849	0.39
Physical Health				−0.215	−3.935	<0.001 **	−0.213	−3.906	<0.001 **	−0.216	−3.939	<0.001	−0.214	−3.893	<0.001 **	−0.214	−3.889	<0.001
Stress							0.086	1.567	0.11	0.097	1.670	0.096	0.081	1.176	0.24	0.061	0.875	0.382
Sleeping Troubles										−0.033	−0.581	0.56	−0.036	−0.638	0.52	−0.042	−0.734	0.46
Burnout													0.032	0.460	0.64	−0.006	−0.086	0.931
Depressive Symptoms																0.110	1.498	0.135
R^2^	0.05			0.08			0.09			0.09			0.09			0.09		
F(df)	24.212 (1.397)			15.481 (1.396)			2.456 (1.395)			0.338 (1.394)			0.212 (1.393)			2.245 (1.392)		
**Men**	**Model 1**			**Model 2**			**Model 3**			**Model 4**			**Model 5**			**Model 6**		
**Absenteeism**	**β**	***t***	***p***	**β**	***t***	***p***	**β**	***t***	***p***	**β**	***t***	***p***	**β**	***t***	***p***	**β**	***t***	***p***
Mental Health	−0.270	−3.341	0.001	−0.213	−2.369	0.01	−0.124	−1.088	0.27	−0.139	−1.200	0.232	−0.133	−1.126	0.26	−0.150	−1.219	0.22
Physical Health				−0.126	−1.399	0.16	−0.105	−1.146	0.25	−0.113	−1.221	0.22	−0.110	−1.186	0.23	−0.111	−1.219	0.22
Stress							0.143	1.264	0.208	0.158	1.376	0.17	0.140	1.092	0.27	0.160	1.188	0.23
Sleeping Problems										−0.069	−0.756	0.45	−0.076	−0.808	0.42	−0.069	−0.716	0.47
Burnout													0.038	0.312	0.756	0.059	0.461	0.645
Depressive Symptoms																−0.069	−0.505	0.614
R^2^	0.06			0.07			0.07			0.07			0.06			0.06		
F(df)	11.160 (1.142)			1.956 (1.141)			1.599 (1.140)			0.571 (1.139)			0.097 (1.138)			0.255 (1.137)		

* *p* < 0.05; ** *p* < 0.001.

**Table 9:** Hierarchical linear regression with mental health, physical health, stress, sleeping troubles, burnout, and depressive symptoms as predictors of absenteeism regarding sexual orientation.

**Heterosexuals**	**Model 1**			**Model 2**			**Model 3**			**Model 4**			**Model 5**			**Model 6**		
**Absenteeism**	**β**	***t***	***p***	**β**	***t***	***p***	**β**	***t***	***p***	**β**	***t***	***p***	**β**	***t***	***p***	**β**	***t***	***p***
Mental Health	−0.251	−5.530	<0.001 **	−0.109	−3.100	0.002 *	−0.109	−1.922	0.05 *	−0.115	−2.013	0.04 *	−0.106	−1.819	0.07	−0.083	−1.337	0.18
Physical Health				−0.206	−4.102	<0.001 **	−0.201	−3.995	<0.001 **	−0.205	−4.053	<0.001 **	−0.203	−4.009	<0.001 **	−0.202	−3.992	<0.001 **
Stress							0.093	1.767	0.078	0.109	1.925	0.05 *	0.076	1.134	0.257	0.061	0.882	0.37
Sleeping Troubles										−0.041	−0.768	0.44	−0.049	−0.904	0.36	−0.056	−1.041	0.29
Burnout													0.059	0.888	0.37	0.034	0.491	0.62
Depressive Symptoms																0.077	1.122	0.26
R^2^	0.06			0.09			0.09			0.09			0.09			0.09		
F(df)	30.584 (1.456)			16.828 (1.455)			3.122 (1.454)			0.590 (1.453)			0.788 (1.452)			1.258 (1.451)		
**LGBTQIA+**	**Model 1**			**Model 2**			**Model 3**			**Model 4**			**Model 5**			**Model 6**		
**Absenteeism**	**β**	***t***	***p***	**β**	***t***	***p***	**β**	***t***	***p***	**β**	***t***	***p***	**β**	***t***	***p***	**β**	***t***	***p***
Mental Health	−0.135	−1.265	0.20	−0.051	−0.416	0.67	−0.008	−0.057	0.95	−0.008	−0.053	0.95	−0.032	−0.195	0.84	0.004	0.020	0.98
Physical Health				−0.171	−1.398	0.16	−0.169	−1.375	0.17	−0.169	−1.367	0.17	−0.173	−1.390	0.16	−0.172	−1.375	0.17
Stress							0.075	0.573	0.56	0.075	0.570	0.57	0.099	0.693	0.49	−0.172	−1.375	0.17
Sleeping Troubles										0.000	0.001	0.99	0.010	0.081	0.93	0.088	0.609	0.54
Burnout													−0.069	−0.451	0.65	0.017	0.140	0.88
Depressive Symptoms																−0.101	−0.599	0.55
**R^2^**	0.007			0.01			0.01			−0.002			−0.012			−0.02		
**F(df)**	1.599 (1.86)			1.954 (1.85)			0.328 (1.84)			0.000 (1.83)			0.203 (1.82)			0.206 (1.81)		

* *p* < 0.05; ** *p* < 0.001.

## Data Availability

The data will be available upon request.

## Notes

1 Psychosocial determinants are factors related to social interactions, working conditions, social support and other aspects of the psychosocial environment that influence an individual’s health and well-being. These factors are associated with a range of health outcomes, including cardiovascular disease and mental health problems (Siegrist & Marmot, 2004).

2 Burnout, especially its central component of emotional exhaustion, is one of the most commonly used indicators of poor mental health in occupational and organizational psychology. This phenomenon results from persistent and chronic occupational stress (Maslach & Leiter, 2016). Given the emotional, physical, and psychological burden it causes, burnout is a significant predictor of heart disease, chronic pain, gastrointestinal problems, depression, and even mortality (Salvagioni et al., 2017).

3 Emotional demands in the workplace refer to factors that require emotional effort due to human interaction (De Jonge & Dormann, 2003).
